# Molecular basis for epitope recognition by non-neutralizing anti-gp41 antibody F240

**DOI:** 10.1038/srep36685

**Published:** 2016-11-09

**Authors:** Neelakshi Gohain, William D. Tolbert, Chiara Orlandi, Jonathan Richard, Shilei Ding, Xishan Chen, Daniel A. Bonsor, Eric J. Sundberg, Wuyuan Lu, Krishanu Ray, Andrés Finzi, George K. Lewis, Marzena Pazgier

**Affiliations:** 1 Division of Vaccine Research of Institute of Human Virology, the University of Maryland School of Medicine, Baltimore, USA; 2 Department of Biochemistry and Molecular Biology, the University of Maryland School of Medicine, Baltimore, USA; 3 Department of Microbiology and Immunology of the University of Maryland School of Medicine, Baltimore, USA; 4 Centre de Recherche du CHUM, Université de Montréal, Montreal, Quebec, Canada; 5 Department of Microbiology, Infectiology and Immunology, Université de Montréal, Montreal, Quebec, Canada; 6 Division of Basic Science of the Institute of Human Virology and Department of Medicine of the University of Maryland School of Medicine, Baltimore, USA; 7 Department of Microbiology and Immunology, McGill University, Montreal, Quebec, Canada

## Abstract

Antibody-dependent cell-mediated cytotoxicity (ADCC) by non-neutralizing antibodies (nnAbs) specific to the HIV envelope (Env) glycoproteins present at the surface of virus sensitized or infected cells plays a role in the effective adaptive immune response to HIV. Here, we explore the molecular basis for the epitope at the disulfide loop region (DLR) of the principal immunodominant domain of gp41, recognized by the well-known nnAb F240. Our structural studies reveal details of the F240-gp41 interface and describe a structure of DLR that is distinct from known conformations of this region studied in the context of either CD4-unliganded Env trimer or the gp41 peptide in the unbound state. These data coupled with binding and functional analyses indicate that F240 recognizes non-trimeric Env forms which are significantly overexpressed on intact virions but poorly represented at surfaces of cells infected with infectious molecular clones and endogenously-infected CD4 T cells from HIV-1-infected individuals. Furthermore, although we detect ADCC activities of F240 against cells spinoculated with intact virions, our data suggest that these activities result from F240 recognition of gp41 stumps or misfolded Env variants present on virions rather than its ability to recognize functional gp41 transition structures emerging on trimeric Env post CD4 receptor engagement.

The HIV-1 envelope (Env) spike (gp120/gp41)_3_ - a trimeric assembly of heterodimers of the transmembrane glycoprotein gp41 and the surface (receptor-binding) glycoprotein gp120 - mediates virus entry to the target cell and is the major target of the humoral anti-viral immune response. Viral entry is initiated by interaction of the envelope spike with the primary receptor on the target cell surface, CD4, and the chemokine co-receptor CXCR4 or CCR5 (reviewed in ref. 1). Although the host receptors’ engagement is mediated by surface glycoprotein gp120 and occurs on the exterior of the Env trimer, it induces a cascade of structural rearrangements of the spike interior with the ultimate goal of activation of the gp41 transmembrane envelope glycoprotein that mediates fusion^1^,^2^.

Transitional epitopes mapped to the gp120 subunit within the first and second constant (C1-C2) region (the A32-like epitopes or Cluster A epitopes^3^, reviewed in refs 4, 5, 6), emerging on virus-sensitized or infected cell surfaces during the conformational rearrangements of Env post CD4 binding, were shown to be targeted by antibodies capable of potent antibody-dependent cell-mediated cytotoxicity (ADCC) without conventional neutralizing activities (refs 3, 7, 8, 9, 10, 11, reviewed in refs 4, 5, 6). Growing evidence points toward a role for these antibodies in protective immunity to HIV-1 during natural infection as well as by vaccination^9^,^10^,^12^,^13^. In contrast, less is known about epitopes localized within the gp41 subunit that could be effective targets for antibodies acting through Fc-mediated effector functions. One such target was identified over a decade ago at the disulfide loop region (DLR) of the principal immunodominant domain (PID) of gp41 (refs 14 and 15 and reviewed in ref. 16) and was shown to be recognized by the monoclonal antibody (mAb) F240^17^. F240 is classified as non-neutralizing/weakly neutralizing antibody capable of Fc-mediated inhibitory activities on macrophages^17^,^18^,^19^, by a mechanism which is not fully understood. The linear gp41 PID sequence recognized by F240 is highly conserved among HIV-1 isolates. F240 was also shown to be broadly cross-reactive and capable of reacting with primary isolates from all clades of HIV-1^17^,^20^.

The binding sites for F240 have been mapped to the loop region within the PID region of the gp41 ectodomain (residues 592 to 606) by mutagenesis and cross-competition studies^17^. However, the structural basis for the F240 paratope-epitope interactions remain unknown. Furthermore, recent studies confirm effective binding of F240 to infectious virions^21^,^22^,^23^,^24^. This effect, as suggested, results from specific recognition of the nonfunctional Env species present on the virus surface^21^,^22^,^23^,^24^. Less is known about the status for these epitopes in the context of transitional and functional Env structures emerging on the target cell during the viral entry process and present on the infected/budding cell surface. Here we elucidate, for the first time, the basis for interaction between F240 and its cognate epitope at the molecular level by describing the 2.5 Å resolution X-ray structure of the complex between the Fab of F240 and the gp41 loop region of the clade B strain BaL. The structure identifies interactions crucial for F240-gp41 binding and maps the F240 epitope to the crown region and vicinities of the DLR. The conformation of DLR bound to F240 is distinct from any other known structures of the gp41 transmembrane envelope glycoprotein. Structural analysis coupled with binding data and ADCC measurements indicate that the F240 epitope is occluded for antibody recognition within the functional trimeric Env expressed on the HIV viral particle or the HIV infected cell surface. However, in contrast to conformational gp120 epitopes of the Cluster A region, which could be induced within the Env trimer upon interaction of gp120 with the CD4 receptor, the exposure of the F240 epitope on virions and the infected cell surface appears to be independent of triggering with membrane CD4.

## Results

### mAb F240 recognizes the oxidized form of the HIV-1 gp41 loop region

**Figure 1:**
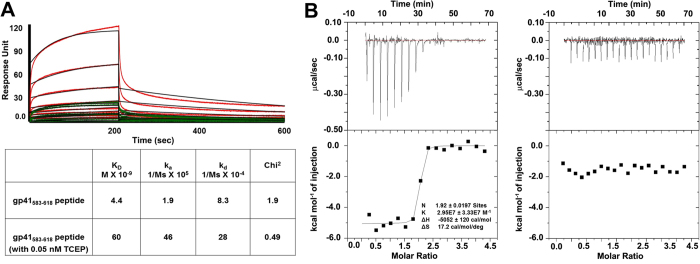
Kinetic and thermodynamic characterization of mAb F240 binding to gp41_583–618_ peptide. (**A**) Surface Plasmon Resonance (SPR) analysis of mAb F240 binding to the gp41_583–618_ peptide (red lines) and the gp41_583–618_ peptide pretreated in reducing (with 0.05 nM TCEP in PBS) conditions (green lines). Sensorgrams were obtained at room temperature for mAb F240 immobilized on a Protein A chip with 0–200 nM concentrations of gp41 peptide passed over the chip. Black and red/green curves correspond to the experimental data (for concentrations of peptide in a range of 3.1–200 nM) and best fit using the BIAevaluation software, respectively. Standard deviations of k_a_, k_d_ and K_D_ for two experiments are shown. **(B**) Isothermal titration calorimetry (ITC) curves for mAb F240 binding to gp41_583–618_ (left) and gp41_583–618_ C698AC605A mutant (right), respectively. The binding isotherms were fitted to a one-site binding model using Origin for ITC version 7.0383 (MicroCal). The fitting yields the stoichiometry (N), the binding constant (K_D_), and the enthalpy (ΔH) of the binding reaction.

The F240 epitope sequence was first described based on pepscan analysis using a set of overlapping peptides (15-mers) to the gp120 and gp41 sequences^17^. These studies mapped the F240 epitope to the immunodominant region of the gp41 ectodomain, residues 592–606. To confirm this initial assessment of the epitope footprint, as well to investigate gp41’s structure when bound to F240, we synthesized the 36-residue peptide spanning the entire loop region of the gp41 immunodominat region corresponding to residues 583–618 (gp41_583–618_). This peptide was evaluated in both oxidized and reduced (with 0.05 nM TCEP) forms for binding to F240 using Surface Plasmon Resonance (SPR) and Isothermal Titration Calorimetry (ITC). As shown in Fig. 1A, F240 recognizes the reduced gp41_583–618_ peptide with approximately 14-fold lower affinity as compared to its oxidized form suggesting that for a steady state binding and generation of a stable F240-gp41 complex, the disulfide bond between Cys^598^ and Cys^604^ is favored. In addition, to exclude the possibility that the reduced gp41_583–618_ peptide could re-oxidize in solution over the course of the reaction confounding the SPR results we substituted the cysteines at position 598 and 604 by alanines to produce a gp41_C598A/C604A_ peptide mutant. We tested binding of F240 to gp41_583–618_ and gp41_C598A/C604A_ peptides in solution by ITC (Fig. 1B). Even though F240 bound very efficiently to gp41_583–618_ (binding constant K_B_ of 2.95  × 10^7^ M^−1^ and the dissociation constant K_D_ of 34 nM) its affinity for gp41_C598A/C604A_ was negligible (Fig. 1B). These results demonstrate that F240 specifically recognizes the oxidized form of the gp41 loop region and that cysteines at positions 598 and 604 (C^598^-C^604^) disulfide bond play a key role in molecular recognition of the gp41 epitope by F240.

### Residues 595–609 of the gp41 ectodomain comprise the F240 epitope

**Figure 2:**
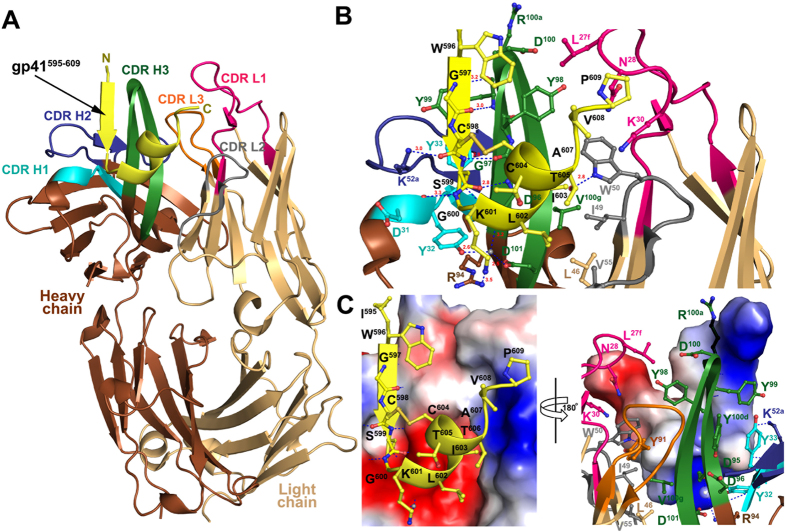
Crystal structure of F240 Fab-gp41_583–618_ complex. (**A**) The ovell structure of F240 Fab-gp41_583–618_ complex shown in ribbon diagram. The light and heavy chains of F240 Fab are shown in light and dark brown, respectively, and the complementarity-determining regions (CDRs) are shown in magenta (CDR L1), grey (CDR L2), orange (CDR L3), cyan (CDR H1), blue (CDR H2), and green (CDR H3). The gp41_583–618_ peptide (residues 595–609 as defined by experimental electron densities) is shown in yellow. (**B**) F240- gp41_583–618_ complex interface. Residues involved in the interactions between F240 Fab and gp41_583–618_ peptide as defined by PISA are shown as sticks. H-bonds are shown as blue dashes and the CDRs are colored as in (A). (**C**) Electrostatic complementarity between the antigen binding site of F240 Fab and gp41_583–618_ peptide. The electrostatic potential is displayed on molecular surface of F240 Fab (left) and gp41_583–618_ peptide (right), which is colored red for acidic, blue for basic and white for apolar. Residues of Fab and peptide involved in the complex interface are shown as ball and sticks and colored as in (**A**).

To further understand the molecular basis for the F240-gp41 interaction and define the structure of the gp41 loop region in the F240-bound state, we determined the crystal structure of the complex of the F240 antigen binding fragment (Fab) and the gp41_583–618_ peptide to 2.5 Å resolution (Table 1 and Fig. 2). The assembly crystallized in monoclinic space group (P21) with two F240- gp41_583–618_ peptide complex copies in the asymmetric unit (ASU) (Table 1). For both copies of the Fab F240 - gp41_583–618_ peptide complex present in the ASU, the electron density map clearly showed a defined density for the residues of the gp41 peptide spanning residues 595–609, which were successfully build to the model. The two copies of complex from ASU were virtually identical with a root square mean deviation (RMSD) of the Cα atoms of 436 residues of the complex of 0.6 Å (Fig. S1).

The overall structure of F240- gp41_583–618_ complex is shown in Fig. 2. In the complex, the gp41 peptide folds into a short β−strand at the N-terminus (residues Trp^596^GlyCys) followed by a turn (Ser^599^GlyLys), an α-helix (residues Leu^602^IleCysThrThr) and a random coil at the C-terminus (residues Als^607^ValPro). The Ser^599^GlyLys turn connects the β−strand to the α-helix and is stabilized by the Cys^598^-Cys^604^ disulfide bond (Fig. 2B,C). The F240 Fab contacts the peptide mainly through complementarity determining regions (CDRs) of its heavy chain with the majority of contacts contributed by CDR H3 (Table S1). The interactive surface that is buried at the F240- gp41_595–609_ interface encompasses a total area of 1584 Å^2^, of which 741 Å^2^ is contributed by the F240 Fab (466 Å^2^ from the heavy chain and 275 Å^2^ from the light chain) and 843 Å^2^ by gp41_583–618_ (Table S1). The heavy chain-peptide interactions are mostly hydrophilic with a number of hydrogen bonds stabilizing the binding interface. The N-terminal β−strand flanking the loop forms an anti-parallel three stranded β-sheet together with the hairpin of the CDR H3 (Fig. 2B). In this region there are four hydrogen bonds formed between the main-chain backbones of the peptide and CDR H3 and one formed for CDR H1. Additionally, main chain-side chain hydrogen bonds are formed between the C^598^S of the peptide and CDR H1 (D^31^Y^32^) and CDR H2 (K^52a^) of the F240 Fab. The interface is further stabilized by a network of water mediated hydrogen bonds involving K^601^ of the S^599^GK turn of the peptide and the CDR H1, CDR H3 and the framework region of the Fab (Fig. S2).

The C-terminal segment of gp41_583–618_ interacts mainly through hydrophobic contacts with residues of light chain CDR L1 and CDR L2. In total 18 hydrophobic contacts as measured by 5 Å cutoff and one side chain-side chain hydrogen bond (between Thr^606^ of the gp41 peptide and Trp^50^ of CDR L2 of the Fab) are formed at the peptide-Fab light chain interface. There is a precise charge complementary fit between the positively charged crown region of gp41_583–618_ peptide, contributed by Lys^601^ and surrounding amino groups of Ser^599^, Leu^602^, and Ile^603^ and the F240 antigen binding site (Fig. 2C).

### F240 and mAb 7B2 recognize very similar epitopes

Several anti-gp41 antibodies have been shown to bind to the linear epitopes within the immunodominant region^14^,^15^,^18^,^25^,^26^, however, to date, the only such antibody characterized at the atomic level with its cognate gp41 epitope is mAb 7B2^27^. Similar to F240, 7B2 was shown to be non-neutralizing but capable of cross-clade recognition of both virus particles and infected cells. In addition it could block infection in macrophages and mediate ADCC against HIV-1 IMC_BaL_ infected primary human CD4+ T cells^22^,^26^,^27^,^28^. Also a 7B2 mutant with enhanced Fc function that was administered passively to rhesus macaques reduced the number of T/F viruses of SHIV-BaL in a high dose intra-rectal challenge study^27^. The Fab of 7B2 was co-crystallized in complex with the gp41 peptide spanning residues 596–606 and the structure was determined at a resolution of 2.7 Å (PDB code: 4YDV).

**Figure 3:**
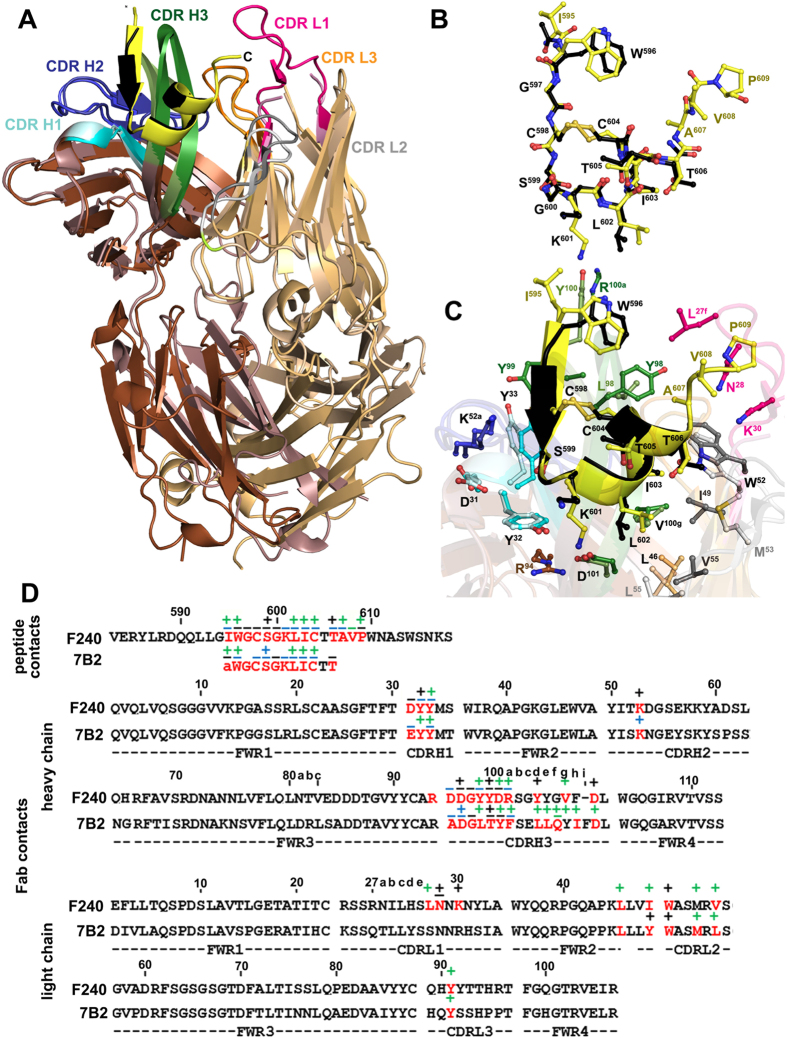
Structural comparison of F240- gp41_583–618_ and 7B2 Fab- gp41_596-606_ complexes. F240 and 7B2 use the same VH3–3.11 and VLκ4.1 germline gene sequences for V_H_ and V_L_ domains, respectively. (**A**) The structure of the F240- gp41_583–618_ complex (dark brown/light brown) was superimposed onto the 7B2 Fab- gp41_596–606_ (dark pink/light pink) (PDB code 4YDV) based on gp41 peptide molecules. The CDRs are shown as dark and light shades as in Fig. 2 for F240 Fab and 7B2 Fab, respectively. The gp41 peptide is shown as a yellow and black ribbon for F240 Fab-gp41_583–618_ and 7B2 Fab-gp41_596–606_ complexes, respectively. (**B**) Superposition of gp41_583–618_ and gp41_596–606_ peptides bound to F240 and 7B2 Fab, respectively. Peptides were superimposed based on main chain atoms. (**C**) The F240 Fab-gp41_583–618_ and 7B2 Fab-gp41_596–606_ binding interfaces. Residues involved in the interactions between F240/7B2 Fab and gp41_583–618_/gp41_596–606_ peptide as defined by PISA are shown as sticks **(D)** Residues in F240 and 7B2 involved in the gp41 peptide binding interface. Buried residues are highlighted red. Main chain (−) and side chain (+) as defined by a 5 Å distance criteria cutoff and colored based on contact type, hydrophobic (green), hydrophilic (blue), or both (black). Framework and CDRs are indicated below the alignment. Only the variable regions of the heavy and light chains for both antibodies are shown.

Structural comparisons of the F240 Fab-gp41_583–618_ and the 7B2 Fab-gp41_596–606_ complexes reveal a remarkable similarity in the conformation of the bound gp41 peptide and the mode of Fab attachment (Fig. 3). The complexes align with a root mean square deviation (RMSD) of 2.63 Å for Cα atoms (as calculated with 113/105 residues of heavy/light chain of the Fab variable domain and 10 residues of the gp41 peptide). In addition, when the bound gp41 peptides (residues 596–606) are superimposed, the positions of their Cα atoms and all atoms differ by an RMSD of 0.343 Å and 1.5 Å, respectively, confirming the very close similarity between the conformation of both the peptide backbone and the side chains involved in Fab binding (Fig. 3B,C). The gp41 peptide bound to F240 is longer by one amino acid residue, Ile^595^, at the N-terminus and by three amino acids, Ala^607^ ValPro at the C-terminus, thus the interface that becomes buried due to the F240- gp41_583–618_ interactions encompasses 1,582 Å^2^ compared to 1,336 Å^2^ buried at the 7B2- gp41_596–606_ complex interface (Table S1). The longer gp41 peptide co-crystallized with F240 Fab reveals new details of the C-terminal fold of the DLR when bound to a PID-specific antibody. This region maintains a random coil conformation with Pro^609^ attached through main and side chain atoms to CDR L1 of the F240 Fab (Fig. 3C).

In both complexes, the interaction is mediated largely by the heavy chain (almost 65–70% of the Fab interaction) and mainly through the residues in the CDR H3 region (Table S1, Fig. 3D), stabilizing the N-terminal β−strand (formed by residues Trp^596^GlyCys and Gly^597^Cys in F240 Fab-gp41_583–618_ and 7B2 Fab-gp41_596–606_ complex, respectively) and the Cys_598_-Cys_604_ bond (Fig. 3B,C). There is a remarkable similarity in how the heavy chain of Fab F240 and Fab 7B2 interact with gp41 with CDR H1 and H2 showing the same conformation and preserving the number and nature of contacts stabilizing the interface (Fig. 3D). In the F240 Fab-gp41_583–618_ complex the CDR H3 of Fab F240 is shifted by 2.36 Å as compared to CDR H3 of Fab 7B2 in the 7B2 Fab-gp41_596–606_ complex to accommodate and stabilize the longer β−strand formed at the N-termini of gp41. Despite the shift, the critical interactions including the hydrogen bond network formed between the hairpin of the CDR H3 and the N-terminal β−strand of the gp41 peptide, are preserved at the 7B2 Fab-gp41_596–606_ interface (Fig. 2B and ref. 27). Unlike the subtle differences in the heavy chain interaction network, prominent changes can be seen in the light chain region with more pronounced differences in conformations of the CDR L2s and CDR L3s. (Fig. 3C). The CDR L1 of F240 Fab binds to the extended C-terminus of the gp41 peptide co-crystalized within the F240 Fab-gp41_583–618_ complex. In the 7B2 Fab-gp41_596–606_ complex CDRL1 is disordered and not resolved in experimental electron densities (Fig. 3C and ref. 27).

### F240 recognizes a unique conformation of the DLR

**Figure 4:**
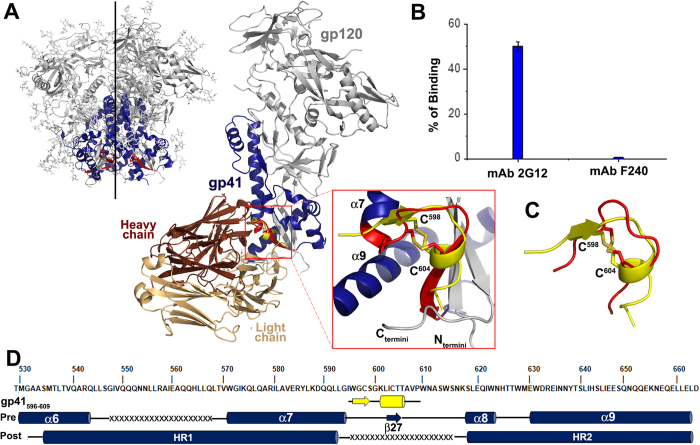
Co-localization of F240 epitope in the context of HIV-1 Env trimer and the solution state of the peptide spanning residues 595–607 of gp41. (**A**) Superimposition of the F240- gp41_583–618_ complex structure onto Cryo-EM structure of a native, fully glycosylated and cleaved HIV-1 Env JR-FL trimer (PDB code: 5FUU). Structures were aligned based on residues 596–609 of the gp41 sequence which is colored yellow for F240- gp41_583–618_ complex and red for the Env JR-FL trimer. The blow-up shows details of comparisons within the 596–609 loop region (**B**) Binding of mAbs 2G12 and F240 labeled with Alexa 647 probe to BG505 SOSIP.664 HIV-1 Env trimer as determined by FCS. (**C**) Superimposition of the gp41_596–609_ peptide from the F240- gp41_583–618_ complex (yellow) and the NMR structure of the peptide spanning residues 595–607 of gp41 (red) (PDB code:1IM7). (**D**) Mapping of the secondary structural elements of gp41 as seen in context of the F240- gp41_583–618_ complex, the pre-fusion state Env JR-FL trimer (PDB code: 5FUU) and post-fusion state (PDB code: 2X7R) onto sequence of the gp41 of BaLisolate. Cylinders represent α-helices, arrows β-strands and the disordered regions are indicated by “x”.

Although several constructs have been developed and studied, also in the context of 3D structures, to understand structural rearrangements of gp41 during viral entry, the conformation of DLR was resolved only in the context of a pre-fusion state of virion-associated, cleaved HIV-1 envelope in a complex with PGT151 Fab^29^ and the free peptide spanning residues 595–607 of gp41 ectodomain, in solution^30^,^31^. To assess if F240 recognizes either of these two known DLR conformations, we superimposed the F240 Fab-gp41_583–618_ complex (based on residues 595–609 of gp41) onto the Cryo-EM structure of PGT151 complexed Env trimer^29^ and a peptide spanning residues 595–607 of gp41 studied in solution by 30,31 NMR (Fig. 4). As shown in Fig. 4A, the F240 epitope maps to a gp41 region of trimeric Env that is buried within the trimer interface engaged directly in interactions with gp120 of the trimer. In addition, structural alignment indicates differences in the secondary structure of the gp41 region forming the nascent F240 epitope within the trimer as compared to the F240 bound state (Fig. 4A, blow up and Fig. 4D). The β−strand (consisting of residues Trp^596^GlyCys) and the α-helix (residues Leu^602^IleCysThrThr) as seen in the F240 Fab-gp41_583–618_ complex are not formed in the Env trimer and are instead replaced by a random coil and short β−strand, respectively. In addition, the β27−strand in the trimer is involved in forming the 3-stranded antiparallel β-sheet with the N- and C-termini of gp120 thus contributing directly to the gp120-gp41 interface. Overall, the observed structural differences and the co-localization of the PID region within the trimer assembly rules out the possibility that the F240 epitope is formed and accessible to antibody recognition in the PGT151-bound Env trimer. In agreement with this observation we were unable to detect the binding of F240 to the soluble preparations of envelope trimer; the BG505 SOSIP.664 gp140 trimer (45) by fluorescence correlation spectroscopy (FCS). F240 was unable to recognize and bind to its epitope on the BG505 SOSIP.664 gp140 trimer whereas mAb 2G12, used as a positive control, bound very efficiently, reaching 50% of binding as determined by fitting the autocorrelation curves (Fig. 4B and S3 refs 23 and 32).

The F240-bound conformation of the PID region also differs significantly from to the structure of the free gp41_595–607_ peptide studied in solution by NMR^30^,^31^. The superimposition (Fig. 4C) reveals significant alterations in the positions of polypeptide main chain atoms and within the secondary structure elements observed in the context of the antibody-bound state that are not present in the unbound state which has several residues in the loop region assuming different conformations. Together, these data indicate that that F240 recognizes a conformation of the gp41 PID region which is buried and not accessible for antibody recognition in the CD4-unliganded PGT151-bound Env trimer and not formed in the gp41 ligand-free peptide in solution.

### F240 effectively recognizes AT-2-inactivated virions

**Figure 5:**
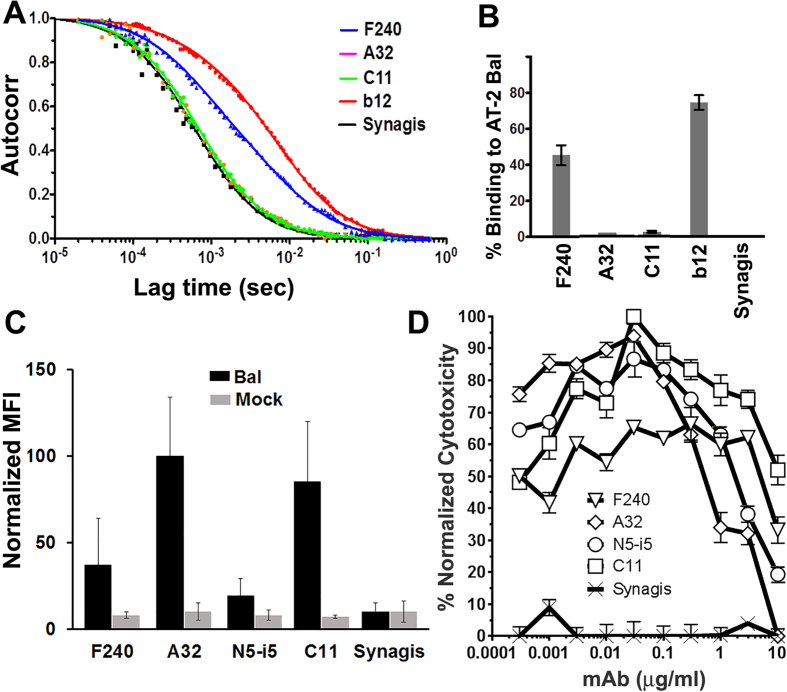
Binding and ADCC activities of mAb F240 against target cells spinoculated with intact HIV virions. (**A,B**) Binding of mAb F240, A32, C11, b12 and Synagis to intact AT-2–inactivated HIV-1 BaL virion as determined by FCS, shown as autocorrelation binding curves and percentage of binding. EGFP-CEM-NKr-CCR5-SNAP cells sensitized with AT-2–inactivated HIV-1 BaL were (**C**) stained for antibody binding and (**D**) ADCC using the modified rapid fluorometric antibody-mediated cytotoxicity assay (RFADCC)^35^. Cluster A CD4i antibodies A32, N5-i5, C11 and Synagis were used as controls. The plot represents a mean of two independent experiments normalized to the average A32 values with error bars showing the range between experiments. Lower than reported in ref. 35 the level of binding of N5-i5 results from its low reactivity with secondary antibody. The curves shown are normalized for plateau cytotoxicity values using the most potent anti-Cluster A mAb C11. An AUC (Area Under Curve) for antibodies tested are (cytotoxicity/μg/ml): 519.2 (F240), 235.6 (A32), 374.8 (N5-i5), 674.5 (C11) and 18.2 (Synagis).

Having established that thegp41 region recognized by F240 is involved in a network of inter-promoter interactions stabilizing the gp41-gp120 interface in the cleaved PGT151-bound Env trimer and most likely occluded for antibody recognition within the properly folded Env trimer we tested if F240 is able to recognize transitional Env structures emerging upon binding of virion to CD4 receptor on the target cell surface. Here we employed a method referred as the virion bound assay^3^ which is designed to detect effector functions of antibodies recognizing transitional epitopes exposed during the earliest stage of viral entry, i.e. the interaction of gp120 of the Env trimer with the host cell receptor, CD4^3^. In this assay EGFP-CEM-NKr-CCR5-SNAP cells are spinoculated with HIV-1 BaL viable virus that was chemically inactivated with aldrothiol-2 (AT-2)^33^ and tested for antibody binding and ADCC in a rapid fluorometric antibody-mediated cytotoxicity assay (RFADCC)^34^,^35^. Using this assay, we previously identified the CD4 inducible (CD4i), gp120 epitope Cluster A, which harbors the most potent ADCC targets in the virion bound assay^3^. The Cluster A epitopes are poorly represented on Env structures expressed on intact virions but become transiently exposed upon interaction of Env with cellular CD4^3^,^7^,^8^,^36^. Figure 5A,B depict binding of F240 and a panel of Cluster A antibodies to free AT-2-inactivated HIV BaL virions in solution (before spinoculation to CD4+ target cells) by FCS. In agreement with previously published data^23^, CD4i Cluster A mAbs A32 and C11 poorly recognized free AT-2-inactivated HIV BaL virions with barely detectable levels of binding (Fig. 5A,B). In contrast but with agreement with previous data using virion capture and FCS methods^21^,^22^,^23^,^24^, F240 bound free, viable AT-2-inactived HIV-1 BaL particles very effectively, reaching 40% of binding which corresponds to almost half of the binding level observed for the CD4 binding site specific mAb b12. The observed lower levels of the F240 binding as compared to mAb b12 and our structural findings, which localize the F240 epitope at the gp120-gp41 interface of virion-bound cleaved Env trimer, indicate, that in the context of AT-2-inactivated HIV-1 virions F240 recognizes non-trimeric/misfolded Env conformations, including gp41 stumps, likely overexpressed at the surface of these AT-2-inactivated viral particles, rather than functional trimeric Env assemblies.

Since in this context F240 attached easily to AT-2-inactivated HIV-1 BaL particles we evaluated whether F240 would recognize target cells spinoculated with these viral preparations. As shown in Fig. 5C,D, F240 effectively recognized cells sensitized with these AT-2-inactivated virions and mediated ADCC in the virion bound assay^3^. Although F240 stained the target cells spinoculated with this viral preparation at levels that were approximately half of the staining detected for anti-Cluster A antibodies (Fig. 5C) it afforded effective ADCC as shown by an AUC (Area Under Curve) of 519 cytotoxicity/μg/ml and a plateau cytotoxicity of 66.4% (Fig. 5D). This places F240 only slightly less potent than the Cluster A mAbs in terms of both AUC value and plateau cytotoxicity (an average 428 ± 224 (SD) cytotoxicity/μg/m AUC and an average plateau cytotoxicity of 93.5 ± 6.6.% (SD)) using this ADCC assay. However, there is a clear distinction between F240 and anti-Cluster A antibodies. Whereas the ADCC activities of Cluster A mAbs against virion-sensitized cells result from recognition of transitional epitope structures emerging on trimeric Env during conformational transitions after CD4 engagement^3^,^7^,^8^ as anti-Cluster A mAbs poorly recognized free AT-2-inactivated HIV BaL virions (Fig. 5A,B), F240 most likely binds the same Env structures, overexpressed on AT2-inactivated HIV BaL particles, which are now attached to target cells. This scenario is supported by significantly lower levels of binding of F240 to the virion bound cells as compared to anti-Cluster A antibodies, that in this context only recognize the properly folded, cleaved and cellular CD4-triggered Env trimers.

### F240 poorly recognizes envelope targets expressed on HIV-1 infected cells

**Figure 6:**
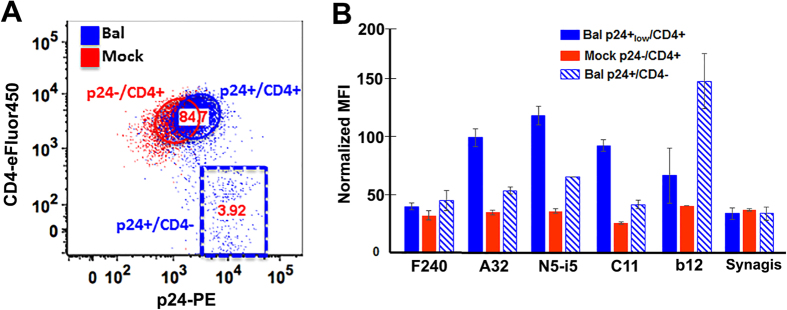
Binding of mAb F240 to EGFP-CEM-NKr-CCR5-SNAP cells infected with BaL infectious molecular clone. (IMC) EGFP-CEM-NKr-CCR5-SNAP were infected with IMC BaL virus and evaluated 5-days post-infection for **(A)** intracellular p24, cell surface levels of CD4 and **(B)** binding of: mAb F240; Cluster mAbs A32, N5-i5 and C11; the CD4-binding site specific mAb b12 and Synagis. Panel A shows the gating strategy for EGFP-CEM-NKr-CCR5-SNAP Bal-infected (Blue gates) and Mock (Red gate) cells, previously defined as GFP+ and L/D dye- cells. For BaL-infected cells two gates were delineated: p24+/CD4− and p24+_low_/CD4+; and one for Mock p24−/CD4+. The plot represents a mean of two independent experiments normalized to the average A32 values with error bars showing the range between experiments.

Having established that F240 binding to AT-2-inactivated HIV virions and target cells spinoculated with these viral preparations likely results from recognition of misfolded Env largely overrepresented on these viral preparations we asked what was the status of the F240 epitope in the context of Env structures present at the surfaces of infected cells. We tested the binding of F240 to populations of EGFP-CEM-NKr-CCR5-SNAP cells after 5-days post-infection with a replication competent HIV-1 BaL infectious molecular clone. As described in ref. 35 this system is designed to infect target cells by cell-free virus or cell–cell virus spread, thus it consists of a dynamic mix of p24 positive cells which are productively infected and have downregulated cell surface CD4 (p24+/CD4− cells) and cells that have viral particles present at their surface but have not been productively infected as illustrated by the absence of CD4 downregulation (p24+_low_/CD4+ cells). Figure 6 shows the binding of F240 to p24+_low_/CD4+ and p24+/CD4− populations, identified by the gating strategy illustrated in Fig. 6A. A panel of anti-Cluster A nnAbs (mAb C11, A32 and N5-i5), known to recognize CD4 inducible epitopes within the inner domain of gp120, and the CD4-binding site specific antibody b12, known to recognize the CD4 un-triggered Env trimer, were used as controls. As expected, the reference anti-Cluster A nnAbs, specific for CD4i epitopes buried in the Env trimer interface^3^,^7^,^8^,^11^, did not recognize mock-infected cells (p24−/CD4+) and productively-HIV-1infected cells with downregulated CD4 (p24+/CD4− cells) but very effectively stained cell populations preserving high levels of cell surface CD4, in agreement with a recent report^37^. These cells are barely detected by the anti-p24 antibody (p24_low_/CD4+) (Fig. 6B) and perhaps result from the recognition of incoming viral particles in an early or non-productive infectious phase. On the contrary, mAb b12 effectively recognized the p24+/CD4− cell population. Interestingly, F240 showed comparable low levels of binding to both p24+/CD4− and p24+_low_/CD4+ cells. Thus, exposure of the F240 epitope on the surfaces of infected cells in this assay format is low, though possibly only slightly above background. Further, these studies suggest that the F240 epitope is not stably exposed at the surface or either p24+low/CD4+ and p24+/CD4− cells, in the context of ongoing viral replication.

**Figure 7:**
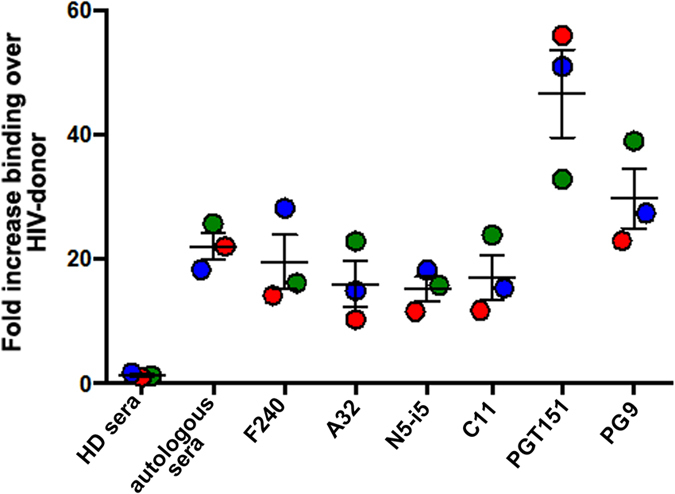
Binding of F240 to the surface of endogenously-infected CD4 T cells. Endogenously-infected CD4 T cells from 3 HIV-1-infected individuals were stained for binding using F240; anti-Cluster A CD4i antibodies A32, N5-i5 and C11 and quaternary epitopes of Env trimer PGT151 and PG9. Before staining, infected CD4 T cells were characterized for p24 staining and cell surface levels of CD4 as shown in Fig. S4C.

This possibility was also tested using “endogenously” infected CD4+ T cells isolated from HIV-1 infected donors. As shown in Fig. 7, the F240 epitope was only weakly detected at the surface of endogenously-infected CD4 T cells isolated from PBMCs obtained from three viremic HIV-1-infected individuals. As shown in Fig. S4, productively-infected (p24+) CD4 T cells retained very low levels of cell surface CD4 staining suggesting that these viruses have functional Nef and Vpu proteins able to downregulate the CD4 receptor. These cells were only weakly recognized by mAbs specific for Epitope Cluster A, indicating that the Env trimer remains in its native state, which is also indicated by the binding of PGT151 and PG9. Notably, the levels of PGT151 and PG9 binding are in agreement with the stoichiometry that these two mAbs are known to recognize trimeric Env (a stoichiometry 2 and 1 for PGT151 and PG9, respectively^38^,^39^). Collectively, these results suggest that the F240 epitope, along with Epitope Cluster A, are poorly exposed at the surface of HIV-1 infected cells unless sufficient cell surface CD4 remains to trigger exposure of the Epitope Cluster A structures, and, even in that case, the F240 epitope either remains buried in the trimer^11^ or it is only transiently exposed to the solvent.

## Discussion

During viral entry, the HIV-1 envelope glycoprotein gp41 traverses at least four sequential conformational states leading to fusion of the viral and target cell membranes. These four conformational states are as follows. Conformational state 1 (CS1) is a metastable, native ‘high-energy’ state that corresponds to the native Env trimer^40^,^41^,^42^,^43^,^44^. Conformational state 2 (CS2) is the pre-fusion CD4-bound state that exposes the co-receptor binding site^44^,^45^. Conformational state 3 (CS3) is the “pre-hairpin” intermediate that is thought to occur upon co-receptor binding to the CD4-bound state^41^,^42^,^46^. Conformational state 4 (CS4) is the post-fusion, highly stable “six-helix bundle”^41^,^42^,^43^,^47^,^48^. The recent wealth of high-resolution structural information emerging from crystallographic and Cryo-EM studies^29^,^40^,^49^,^50^ provides an increasingly clear picture of the gp41 ectodomain, including the DLR and PID regions, for CS1, the native Env trimer. By contrast, there is relatively little information about the DLR and PID sub-regions of the gp41 ectodomain for the other conformational states that occur after receptor triggering. While a number of studies have used synthetic peptides or short recombinant gp41 proteins to mimic CS3 and CS4^40^,^41^, including atomic structures, there is no data about the conformation of the DLR in the context of these conformational states. All available structures consist of the gp41 ectodomain core with the loop region either omitted or missing in the final structural model due to a lack of interpretable densities^43^,^47^,^51^. The only available structures of the DLR studied in the context of the disassembled Env trimer are the solution structure of the SIV gp41 ectodomain mutant (with loop cysteines mutated to alanines) determined by NMR spectroscopy^52^ and structures of peptides spanning residues 595–607 of HIV-1 gp41 in the solution state^30^,^31^.

The broadly reactive, non-neutralizing F240^53^,^54^ was isolated from an HIV infected individual in the late 1990’s^17^. It has been shown that F240 displays Fc-mediated inhibitory activities via macrophages (against clade B HIV-1 BaL and BX08 and clade C TV-1 isolates)^18^ and human monocyte-derived macrophages (against the clade B HIV-1 SF162 strain)^19^. Inhibitory activities of F240 are thought to be mainly due to Fc-mediated phagocytosis although the exact mechanism by which F240 exhibits its full inhibitory effect is not fully understood. In particular, it remains uncertain as to whether F240 is capable of affecting infection through ADCC. In agreement with its poor ability to recognize HIV infected cells, F240 was recently found to be unable to mediate ADCC against primary HIV-1 SF162-infected CD4+ T cells with natural killer (NK) cells as effectors^19^ or CEM.NKr cells infected with NL4.3 GFP coding for the ADA Env or transmitted/founder CH77 infectious molecular clones with human PBMCs as effectors^11^.

The F240 binding site was mapped into the linear epitope of the DLR of the gp41 ectodomain with no structural details about the conformation of this region^17^. Our data show that F240 preferentially recognizes the oxidized form of the DLR and for the first time describe the F240 epitope structure. Structural analysis indicates that the F240 epitope shares extensive structural features with another PID specific antibody 7B2^27^. F240 and 7B2 recognize a unique conformation of the PID loop that is conformationally distinct from this region in the pre-fusion state Env trimer and the solution structure^29^ of the unbound peptide consisting of residues 595–607 of the gp41 ectodomain^28^. In addition, the region recognized by F240 is buried at the PGT151-bound Env trimer interface thus not accessible for antibody interaction in properly folded CD4-untriggered Env trimers. This evidence disagrees with recent F240 capture studies indicating that F240 is very efficient in binding to both infectious and non-infectious virions, with quantities of mAb bound several fold greater than those observed for neutralizing antibodies specific for functional trimers^20^,^21^,^22^,^23^,^55^. The strong binding of F240 to virions has been attributed to the presence of large fractions of non-functional Env species on the surface of both infectious and non-infectious virions. In this context, F240 is proposed to bind to gp41 stumps, misfolded or partially folded Env variants, including oligomeric assembles with one or two gp120 protomers missing and gp120/gp41 heterodimers alone^20^,^21^,^22^,^23^,^55^. In light of our structural comparisons, showing significant dissimilarities between conformations of the PID region within the Env trimer, as a free peptide in solution and in the F240-bound state, in this scenario one assumes that the F240 epitope structure results from the antibody driven induced fit on the polypeptide backbone of the exposed gp41 regions or conformational selection of the misfolded structures remaining on the viral surface.

Although as we show here and it was shown previously^20^,^21^,^22^,^23^,^55^, F240 effectively binds to non-functional envelope forms expressed on free virions, our data indicate that it poorly recognizes envelope conformations present at the surface of cell populations infected with replication competent HIV-1 infectious molecular clones which have or have not downregulated cell surface CD4 after several days post-infection. This data agrees with studies of others with infected cell lines^11^,^56^, including cells infected with infectious molecular HIV-1 clones harboring wild-type or defective *nef* and *vpu* genes^11^, as well as cells infected with lab-adapted or primary HIV-1 and SHIV isolates^11^,^56^. Furthermore we found F240 to be inefficient in recognizing both the EGFP-CEM-NKr-CCR5-SNAP cell line as well as endogenously-infected primary CD4+ T cells with quantities of mAb bound in a range similar to the anti-gp120 CD4i antibodies of the Cluster A region. Of note, under our reactivation protocol, no more than 7% of the endogenously-infected primary CD4+ T cells were p24+ after 7 days of culture (Fig. S4C), suggesting that the absence of F240 recognition is not due to late stage infection. Finally, when infected EGFP-CEM-NKr-CCR5-SNAP cell populations were gated based on the cell surface CD4 levels, F240 was stained at similar levels in the populations with or without CD4 present. This observation indicates that the Env interaction with cellular CD4 does not play an important role in F240 epitope exposure at the infected cell surface. In this context, it is possible that F240, as in viral particles, recognizes non-trimeric Env variants present at the surface of infected cells or viral particles. Furthermore our data indicate that the ability of F240 to eliminate the target cells spinoculated with intact HIV BaL virions by ADCC in our virion bound RFADCC assay^35^ most likely results from its ability to recognize non-trimeric/misfolded Env variants present on free particles and not to binding to functional transitional Env structures induced by the cellular CD4 binding. This is in sharp contrast to Cluster A antibodies where epitopes are induced on the Env trimer only upon binding to the CD4 receptor.

Growing evidence suggest that FcR-effector functions of antibodies without classical direct neutralization play a role in protection against HIV-1 infection *in vivo*. The FcR-mediated activities of antibodies may involve ADCC but also other anti-viral mechanism requiring recognition of epitopes directly at the virial surface such as antibody-dependent phagocytosis (ADCP), antibody dependent cellular viral inhibition (ADCVI) or complement mediated virolysis (reviewed in refs 57, 58, 59). mAbs F240 and 7B2 were recently used in three passive transfer studies in NHPs to provide evidence of the role of non-neutralizing antibodies in protection against high-dose SHIV challenge^19^,^24^,^27^. Although these NHP passive transfer studies failed to afford protection against SHIV acquisition, post-infection control of viremia and a reduced number of transmitted variants were observed with F240 and 7B2, respectively, indicating that anti-PID nnAbs impacted the transmitted virus by mechanisms other than classical neutralization^19^,^24^,^27^. Indeed, our studies confirm that F240 recognizes aberrant/misfolded and most-likely non-functional Env structures largely present on the surface of free virions making it incapable of direct neutralization due to the lack of binding to properly folded functional Env spikes. However, since F240 shows excellent properties in recognizing native virions it may act through other anti-viral Fc-effector mechanisms that require effective virus coating such as ADCP or ADCVI. Indeed, very recent studies provide direct evidence of 7B2 being capable of antibody-mediated internalization of HIV-1 virions, a mechanism in which antibody recognizes a virus and engages Fc receptors on phagocytes to cause internalization^60^. The antibody-mediated internalization of HIV-1 virions was shown to not be dependent on the capability of antibody to directly neutralize but instead appeared to be a function of antibody specificity and isotype/subclass^60^. Finally our data indicating persistence of F240 epitope targets on the surface of virus attached to target cells point toward a possibility that F240 may contribute to the virial clearance of cell targets during HIV-1 entry through an ADCC mechanism.

It is important to note that our studies identify the molecular basis for the F240 epitope in the context of two out of four steps identified during viral entry, including CS1, a metastable, ‘high energy’ conformation on the free virus particle, and CS2, the CD4-bound conformation. We cannot rule out the possibility that the F240-bound DLR conformation represents a functionally relevant structure of the gp41 DLR intermediate occurring later during viral entry. In this context the F240-bound PID loop structure may represent a transitional structure of the DLR in CS3, the pre-hairpin intermediate or CS4, the highly stable “six-helix bundle” post-fusion structure. Studies using specific inhibitors for these steps are underway to clarify the aforementioned possibilities.

## Materials and Methods

### Production and purification of F240 monoclonal antibody

F240 was generated as previously described^3^ using molecular clones of the heavy and light chains provided by Dr. Yongjun Guan. F240 Fabs were prepared from purified IgG (10 mg/ml) by proteolytic digestion with immobilized papain (Pierce, Rockford, IL) and purified using protein A (GE Healthcare, Piscataway, NJ), followed by gel filtration chromatography on a Superdex 200 16/60 column (GE Healthcare, Piscataway, NJ).

### gp41 peptide synthesis and folding

The amino acid sequence of the 36-residue peptide of the sequence 583–618 of gp41 (based on the clade B BaL sequence) is gp41_583–618_: VERYLRDQQL^10^ LGIWGCSGKL^20^ ICTTAVPWNA^30^ SWSNKS. The peptide was synthesized on an ABI 433A automated peptide synthesizer using the optimized HBTU activation/DIEA *in situ* neutralization protocol developed by Kent and colleagues for Boc-chemistry solid phase peptide synthesis (SPPS). After cleavage and deprotection in HF, crude product was precipitated with cold ether and purified to homogeneity by preparative C18 reversed-phase HPLC to afford reduced peptide. The molecular masses were confirmed by electrospray ionization mass spectrometry (ESI-MS). Mass: observed 4082.0 Da, calculated 4081.6 Da. The reduced peptide was dissolved at 0.4 mg/ml in 1 M GuHCl containing 20% DMSO (v/v) for disulfide formation. After 2 hours, reaction was completed and purified with RP-HPLC to afford oxidized peptide. Mass (ESI): observed. 4079.9 Da, calculated. 4079.6 Da. The gp41_583–618_ C598AC604A peptide mutant was synthetized using the same protcol developed by Kent *et al.* for Boc-chemistry SPPS and mutating the two cysteine at position 618 and 605 to alanine.

### Surface Plasmon Resonance (SPR)

The binding affinity and kinetics of F240 for gp41_583–618_ peptide and gp41_583–618_ peptide pretreated to reduce C^598^-C^604^ disulfide bond was assessed by Surface Plasmon Resonance on a Biacore T-100 (GE Healthcare) at 25 °C. The reduced gp41_583–618_ was generated by treating the oxidized form of the same peptide with 0.05 M of TCEP. Protein A was first immobilized onto the second of the two flow cells on a CM5 chip to ~3000 response units (RU) and the first flow cell blocked with a standard amine coupling protocol (GE Healthcare). F240 IgG to be evaluated was then captured onto the second flow cell by flowing a 5–10 nM solution of mAb at 10 μl/min flow rate for 30 seconds. The antibody concentration was varied to give a RU in the range of 120 to 150. Varying concentrations (0–200 nM) of the oxidized and reduced forms of the peptide were then passed over both flow cells at a flow rate of 30 μl/min for 200 seconds and allowed to dissociate by passing buffer over both cells at the same flow rate for 800 seconds. The cells were regenerated with a 30 second injection of 0.1 M glycine pH 3.0 with a flow rate of 100 μl/min and the antibody reloaded onto the second flow cell for each peptide concentration. Blank sensorgrams were obtained by injection of HBS-EP buffer (10 mM HEPES, pH 7.4, 150 mM NaCl, 3 mM EDTA, and 0.05% surfactant P-20) in place of the peptides. Sensorgrams of the concentration series (flow cell two minus one) were corrected with corresponding blank and the kinetic constants (association rates (*k*_*a*_), dissociation rates (*k*_*d*_), and affinity constants (*K*_D_)) were determined using a 1:1 Langmuir model of binding with the BIAevaluation software (GE Healthcare).

### Isothermal Titration Calorimetry (ITC)

The ITC experiments were performed using an ITC200 system (Micro-Cal) as previously described by Bradshaw *et al.*^61^, at 25 °C in a 1X PBS buffer pH 7.4. F240 was dialyzed against this buffer overnight before the experiment. The peptides were dissolved in the final dialysis buffer. A typical experiment had F240 in the syringe (111 μM in 1X PBS pH 7.4) and gp41_583–618_ or gp41_583–618_ C598AC604A peptide in the cell (11 μM in 1X PBS pH 7.4). Titrations were performed at 25 °C with 17 injections of 2.42 μl aliquots, with 210–240 second intervals between injections. Heats of dilutions were measured and subtracted from each data set. Data were corrected for the heat of dilution and fitted using a nonlinear least-squares routine using a single-site binding model with Origin for ITC version 7.0383 (MicroCal). Raw data for representative experiments are included in Fig. 1B.

### Crystallization of the F240- gp41_583–618_ peptide complex

The F240- gp41_583–618_ peptide complex was prepared by mixing 1:1.5 molar ratio of F240 Fab and gp41_583–618_ peptide, purified using size exclusion chromatography (Hiload 26/60 Superdex S200 prep grade, GE Healthcare in 0.35 M NaCl, 5.0 mM Tris pH 7.2) and concentrated to approximately 9 mg/ml for crystallization experiments. Initial screening was performed using the Art Robbinson crystallization robot for the sitting drop diffusion method with commercially available crystal screens from Hampton and Qiagen. Conditions that produced micro crystals with robotic screening were further optimized by hand using the hanging drop method with respect to the protein concentration, precipitant concentration and pH to increase the size and improve the appearance of the crystals obtained. Two conditions comprising of 25% PEG 3350, 15% isopropanol, 0.2 M ammonium citrate pH 4.5 and 0.01 M magnesium chloride hexahydrate, 0.05 M HEPES pH 7.0, 1.6 M ammonium sulfate gave diffraction quality crystals. Data sets were collected for the crystals from both the conditions however, the condition that gave the crystals with a better data set were obtained from the drops containing 0.5 μl of protein mixed with equal volume of the reservoir solution containing 25% PEG 3350, 15% Isopropanol, 0.2 M ammonium citrate pH 4.5.

### Data Collection, Structure Solution and Refinement

Crystals were flash frozen in liquid nitrogen after briefly soaking in the crystallization condition plus 20% MPD prior to data collection. Diffraction data were collected at the Stanford Synchrotron Radiation Light Source (SSRL) BL7–1 beam line on an ADSC Quantum 315 area detector. The data was processed and scaled with HKL2000 package. The assembly crystallized in monoclinic space group (P21) with the unit-cell parameters a = 49.3, b = 60.5, c = 169.2 Å and β = 94.1° with two F240- gp41_583–618_ peptide complex copies in the asymmetric unit (ASU) (Table 1). The structure was solved by molecular replacement with Phaser from CCP4i suite^62^ based on the coordinates extracted from the structure of N5-i5 Fab (PDB Code: 3TNN ref). The model was refined using Refmac and the structure was completed manually using COOT^63^. Molecular graphics were generated using PyMol.

### Structure validation and analysis

The quality of the final refined models was monitored using the program MolProbity^64^. Structural alignments were performed using the Dali server and the program lsqkab from the CCP4 suite. The PISA webserver was used to determine contact surfaces and residues. All illustrations were prepared with the PyMol molecular graphic suite (DeLano Scientific, San Carlos, CA, USA). The Ramachandran plot obtained by the validation program “MolProbity” shows 92.4% of the total amino acids in the most favored region 5.5% and 2.1% residues in the generously allowed and disallowed regions, respectively.

### Fluorescence correlation spectroscopy (FCS) measurements

mAbs F240, 2G12 A32, C11, b12 and Synagis (MedImmune) were labeled with Alexa 647 probe (Invitrogen mAb labelling kit) for FCS experiments. Briefly, the Alexa Fluor 647 reactive dye has a succinimidyl ester moiety that reacts efficiently with primary amines of MAb to form stable dye-protein conjugates. The dye labeled mAb was purified using 10 KDa spin columns. Purified Alexa-647 labeled MAbs were quantified by a UV-visible (UV-vis) spectrometer (Nanodrop 2000). Dye-to-protein ratios were determined to be ~3 by measuring absorbance at 280 nM (protein) versus 647 nM (dye). FCS measurements were performed in a confocal microscope (ISS Q2). ISS VistaVision software was used to analyze the FCS data to assess the *in vitro* binding of mAbs to BG505 SOSIP.664^65^ and AT-2–inactivated HIV-1_BaL_ virions. To facilitate the formation of complexes, we incubated mAbs (5 μg/ml) with 50 μg/ml of SOSIP or 10 μg/ml of AT-2–inactivated HIV-1_BaL_ virions for 90 minutes at 37 °C. We determined the translational diffusion coefficients of Alexa 647 labeled mAbs and the corresponding complexes with BG505 SOSIP or virion. The FCS measurements and analyses were performed similar to previously reported^23^,^32^.

### RFADCC assay

The ADCC activity of F240, A32, N5-i5, C11 and Synagis (MedImmune) was measured using the modified rapid fluorometric antibody-mediated cytotoxicity assay (RFADCC) that permits high-throughput processing of samples as described in ref. 35 using target cells spinoculated with intact HIV virions (virion bound assay) or infected by cell-free virus. In brief, EGFP-CEM-NKr-CCR5-SNAP target cells were first stained with the SNAP-Surface Alexa Fluor 647 dye, washed twice and spinoculated with HIV-1 BaL AT-2 inactivated virus at 2000 RPM for 2 h at 12 °C. Subsequently, AT-2 BaLsensitized double-stained targets were washed twice with cold R10 medium and then subjected to testing for antibody binding (detected with PE-labeled mouse anti-human IgG secondary antibody, BD Bioscence cat. 555787) and RFADCC assay, following the previously described protocol^35^. Samples were collected on a BD Fortessa Special Order instrument (BD Biosciences) and analyzed using FlowJo software (Tree Star, Ashland, OR). The HIV-1BaL AT-2 inactivated virus was generously provided by Dr. Jeff Lifson, NCI, Frederick. The ADCC data represent the typical results obtained in two independent experiments done in duplicate and the bars indicate the range of the values of cytotoxicity.

### Infection of EGFP-CEM-NKr-CCR5-SNAP cells infection with an HIV-1_BaL_ molecular clone and cell-surface staining

EGFP-CEM-NKr-CCR5-SNAP cells were spinoculated for 2 hr at 2000 RPM at 12 °C in 96-well U-bottom plate (5 × 10^5^ cells/well) with 240 ng of IMC BaL virus (control cells were incubated without virus), as measured by HIV-1 p24 antigen capture ELISA. Afterward, the viral inoculum was diluted 1:2 in R10 medium containing G418 1.5 mg/ml, and cells and virus were placed into one well of a 12-well flat-bottom plate. The cells were then cultured adding fresh medium every 2 days. At 5 days post-infection, EGFP-CEM-NKr-CCR5-SNAP cells were harvested and washed twice with R10 medium and stained with F240, A32, N5-i5, C11, b12 and Synagis (MedImmune) mAbs labeled with Alexa Fluor-647, Live/Dead Fixable Near-IR Dead Cell Stain (Molecular Probes) and with 5 ul of (eFluor 450)-conjugated mouse anti-CD4 OKT4 mAb (eBioscience) for 30 min at RT. After a wash, cells were then fixed and permeabilized using the Cytofix/Cytoperm Kit (BD-PharMingen, San Diego, Calif.) for 20 min at 4 °C. Subsequently, permeabilized cells were washed once with the buffer provided by the manufacturer, resuspended and stained for 30 min at RT with 5 μl (PE)-conjugated mouse anti-p24 mAb (KC57-RD1; Beckman Coulter, Inc.). After two additional washes, HIV-1- or mock were fixed in PFA 2% analyzed with an LSRII Fortessa flow cytometer (BDBiosciences) and data analysis was performed with FlowJo software (Tree Star,Inc., San Carlos, Calif.). The plot represents a mean of two independent experiments normalized to the average A32 values with error bars showing the range between experiments.

### *Ex-vivo* amplification and cell-surface staining of endogenously infected CD4+ T cells

Endogenously-infected CD4 T cells from HIV-1-infected individuals were expanded *ex-vivo in vitro* as previously described^66^. Briefly, primary CD4 T cells were isolated from PBMCs obtained from three viremic HIV-1-infected individuals. Purified CD4+ T cells were activated with PHA-L at 10 μg/ml for 36 hours and then cultured for 7 days in RPMI-1640 complete medium supplemented with rIL-2 (100 U/ml). Viral replication was measured via intracellular staining for p24 (clone KC57-RD1; Beckman Coulter) and cell-surface staining was performed as previously described^10^,^66^. Cells were stained with either sera (1:1000 dilution), 1 μg/ml of mouse anti-CD4 mAb OKT4 (14–0048–82, eBiosciences, San Diego, CA, USA) or 5 μg/ml of mAbs F240, PGT151 (kindly provided by IAVI), PG9, A32 and N5-i5. 1 μg/ml of goat anti-mouse or anti-human Alexa Fluor-647 mAbs (Invitrogen, San Diego, CA, USA) was used as secondary Ab. AquaVivid (Invitrogen, San Diego, CA, USA) was used as a viability dye. The percentage of infected cells (p24+ cells) was determined by gating the living cell population based on the viability dye staining. Samples were analyzed on a LSRII cytometer (BD Biosciences, Mississauga, ON, Canada) and data analysis was performed using FlowJo vX.0.7 (Tree Star, Ashland, OR, USA). Written informed consent was obtained from all study participants, and research adhered to the ethical guidelines of the University of Montreal Hospital Research Centre (CRCHUM) and was reviewed and approved by the CRCHUM institutional review board (ethics committee).

## Additional Information

**How to cite this article**: Gohain, N. *et al.* Molecular basis for epitope recognition by non-neutralizing anti-gp41 antibody F240. *Sci. Rep.* **6**, 36685; doi: 10.1038/srep36685 (2016).

**Publisher’s note:** Springer Nature remains neutral with regard to jurisdictional claims in published maps and institutional affiliations.

## Supplementary Material

Supplementary Information

## Figures and Tables

**Table 1:** Data collection and refinement statistics.

	F240-gp41 complex
Data collection	
Wavelength, Ǻ	0.9753
Space group	P2_1_
Cell parameters	
a, b, c, Å	49.32, 60.49, 169.2
α, β, γ, °	90.0, 94.13, 90.0
complexes/a.u.	2
Resolution, (Å)	168.8–2.5 (2.59–2.55)
# of reflections	96537
Total	33289
Unique	15.8 (95.7)
R_merg_^b^, %	9.4 (1.4)
I/σ	98.8 (98.6)
Completeness, %	2.9 (2.9)
Redundancy	
Refinement Statistics	
Resolution, Å	38.4–2.54
R^c^, %	20.4
R_free_^d^, %	25.9
# of atoms	
Protein	7013
Water	212
Ligand/Ion	153
Overall B value (Å)^2^	
Protein	37.41
Water	49.4
Ligand/Ion	31.5
Root mean square deviation	
Bond lengths, Å	0.010
Bond angles, °	1.397
Ramachandran^e^	
favored, %	92.4
allowed, %	5.52
outliers, %	2.1
PDB ID	5DRZ

Values in parentheses are for highest-resolution shell.

^b^*R*_merge_ = ∑│*/* − <*/*>│/∑*/*, where */* is the observed intensity and <*/*> is the average intensity obtained from multiple observations of symmetry-related reflections after rejections.

^c^*R* = ∑||F_o_│−│ F_c_||/∑│F_o_│, where F_o_ and F_c_ are the observed and calculated structure factors, respectively.

^d^R_free_ = defined by by Brünger.

^e^Calculated with MolProbity.
