# Supplementary material for: Molecular basis for epitope recognition by non-neutralizing anti-gp41 antibody F240
Source: Sci Rep. 2016 Nov 9;6:36685. doi: 10.1038/srep36685 (PMC5101508; doi:10.1038/srep36685)
Supplement: Supplementary Information [file srep36685-s1.pdf]

Molecular basis for epitope recognition by non-neutralizing anti-gp41 antibody F240

Neelakshi Gohain[1,2], William D. Tolbert[1,2], Chiara Orlandi[1,3], Jonathan Richard[4,5], Shilei Ding[4,5], Xishan Chen[1,2], Daniel A. Bonsor[1,6], Eric J. Sundberg[1,3,6], Wuyuan Lu[1,2], Krishanu Ray[2], Andrés Finzi[4,5,8], George K. Lewis[1,3], Marzena Pazgier[1,2#]

[1]Division of Vaccine Research of Institute of Human Virology and [2]Department of Biochemistry and Molecular Biology and [3]Department of Microbiology and Immunology of University of Maryland School of Medicine, Baltimore, USA

[4]Centre de Recherche du CHUM and [5]Department of Microbiology, Infectiology and Immunology, Université de Montréal, Montreal, Quebec, Canada,

[6]Division of Basic Science, of Institute of Human Virology and Department of Medicine of University of Maryland School of Medicine, Baltimore, USA

[8]Department of Microbiology and Immunology, McGill University, Montreal, Quebec, Canada,

[#]To whom correspondence should be addressed:

Email: mpazgier@ihv.umaryland.edu

725 West Lombard Street, Baltimore, MD 21201, USA

Tel: (410)706-4780

Fax: (410)706-7583

Supplementary Figures and tables

Table S1. Buried surface area as calculated by the EBI PISA server (http://www.ebi.ac.uk/msd-srv/prot_int/cgi-bin/piserver).

		**Fab F240-gp41**			**Fab 7B2-gp41**		
		**1st copy**	**2nd copy**	**Average**	**1st copy**	**2nd copy**	**Average**
**Buried Surface Area, Å²**	**gp41 total**	**808.7**	**876.3**	**842.5**	**735.3**	**727.1**	**731.2**
	**Heavy chain total**	**462.3**	**470.4**	**466.4**	**425**	**425.9**	**425.5**
	FWR H	4.1`	7.9	6.0	0	0	0
	CDR H1	58	57.7	57.9	42.3	45.1	43.7
	CDR H2	18.8	18.9	18.85	20.5	19.6	20.1
	CDR H3	381.4	385.9	383.7	362.2	361.2	361.7
	**Light chain total**	**255**	**294.6**	**274.8**	**182.8**	**177.7**	**180.3**
	FWR L	52	51.5	51.7	56.8	56.1	56.5
	CDR L1	88.8	133.4	111.2	0	0	0
	CDR L2	101.3	96.6	99.0	112.8	109.5	111.2
	CDR L3	12.9	13.1	13.0	13.2	12.1	12.7
	**Heavy and light chain total**	**717.3**	**765**	**741.2**	**607.8**	**603.6**	**605.7**

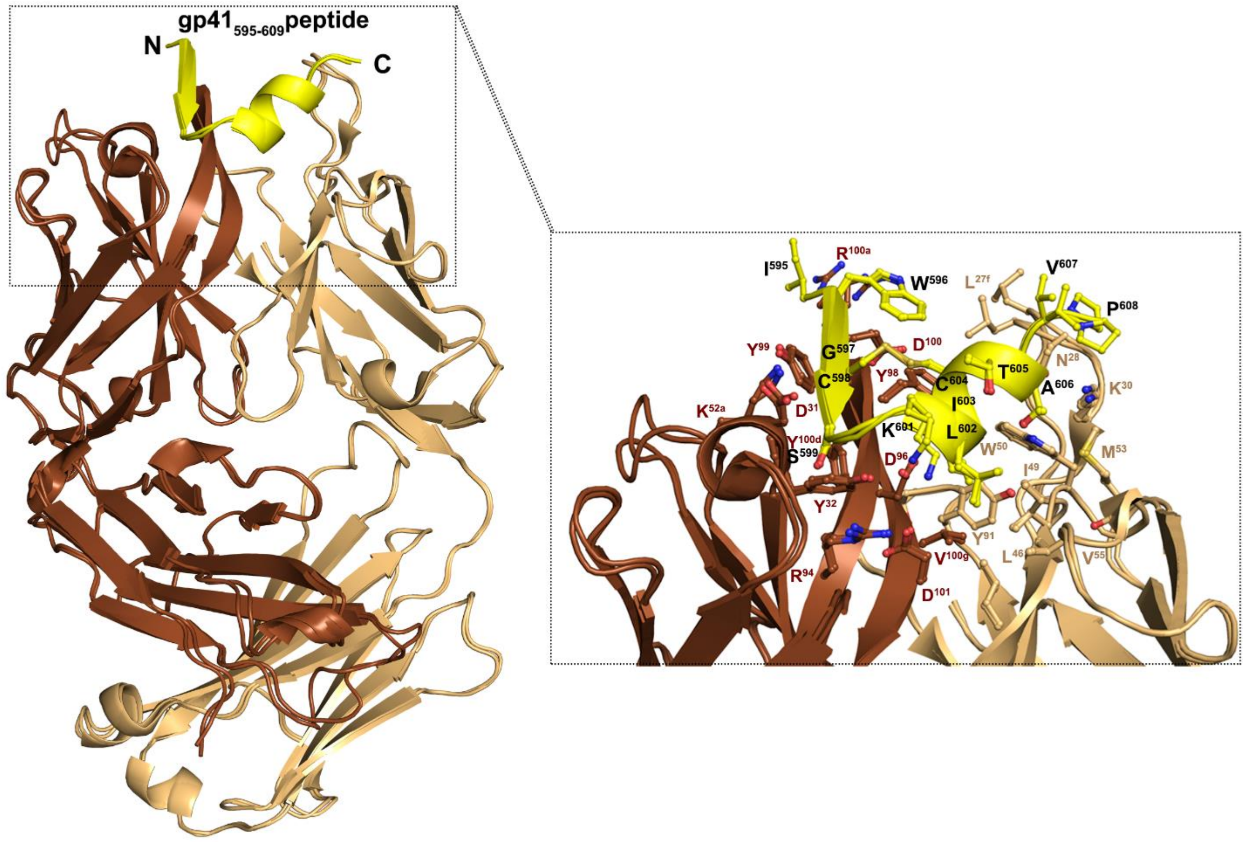

Figure S1: Ribbon diagram of superposition of the two copies of F240 Fab- gp41$_{583\text{-}618}$ complex from the asymmetric unit of the crystal. The blow up shows the F240 Fab-gp41$_{583\text{-}618}$ binding interface with residues contributing to the interface shown as sticks. Overall structures of these copies are very similar with the root mean square deviation (RMSD) of equivalent Cα atoms of 0.61 Ǻ for the complex, 0.62 Ǻ for Fab molecules and 0.47 Ǻ for gp41$_{583\text{-}618}$ peptide.

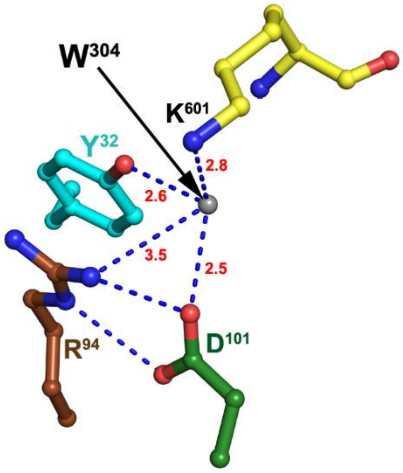

Figure S2: Network of water mediated H-bonds at the F240Fab- $gp41_{583-618}$ interface.

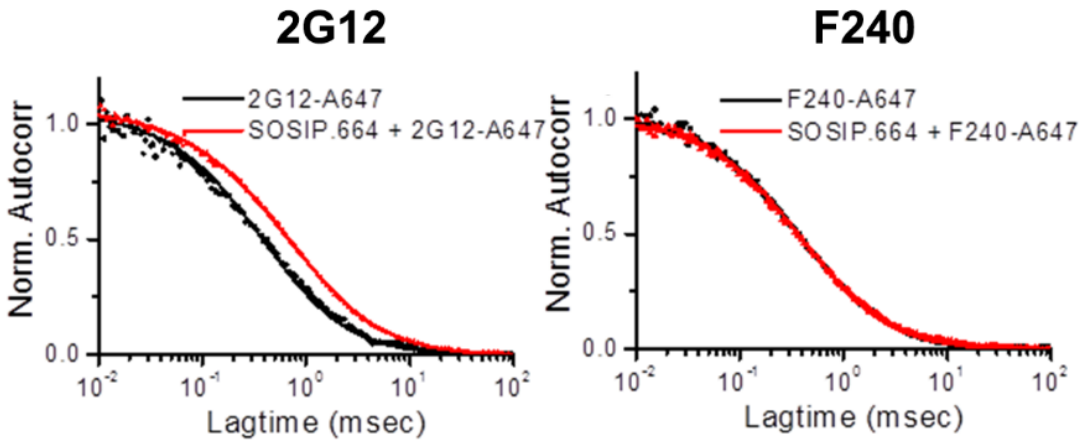

Figure S3: Autocorrelation curves of Alexa 647 labeled mAb 2G12 and F240 in the presence or absence of SOSIP. From the FCS measurements, the translational diffusion coefficient of Alexa 647 labeled 2G12 or F240 is determined to be 60±3 μm^2/sec after fitting the autocorrelation curves with a diffusion model. The diffusion coefficient of F240 remain unchanged even after incubating with BG505 SOSIP suggesting F240 does not bind to SOSIP which is in accordance with the binding profile of F240 to SOSIP as reported earlier. To confirm the binding profile of F240 to SOSIP in solution, we included Alexa 647 labeled 2G12 as a positive control. The diffusion coefficient of Alexa 647 labeled 2G12 has been significantly decreased upon binding to BG505 SOSIP. The diffusion coefficient for this 2G12-SOSIP complex is determined to be 20±2 μm^2/sec. By fitting the autocorrelation curve of 2G12-SOSIP complex, the fraction of bound 2G12 is determined to be 50% as shown in Figure 4B.

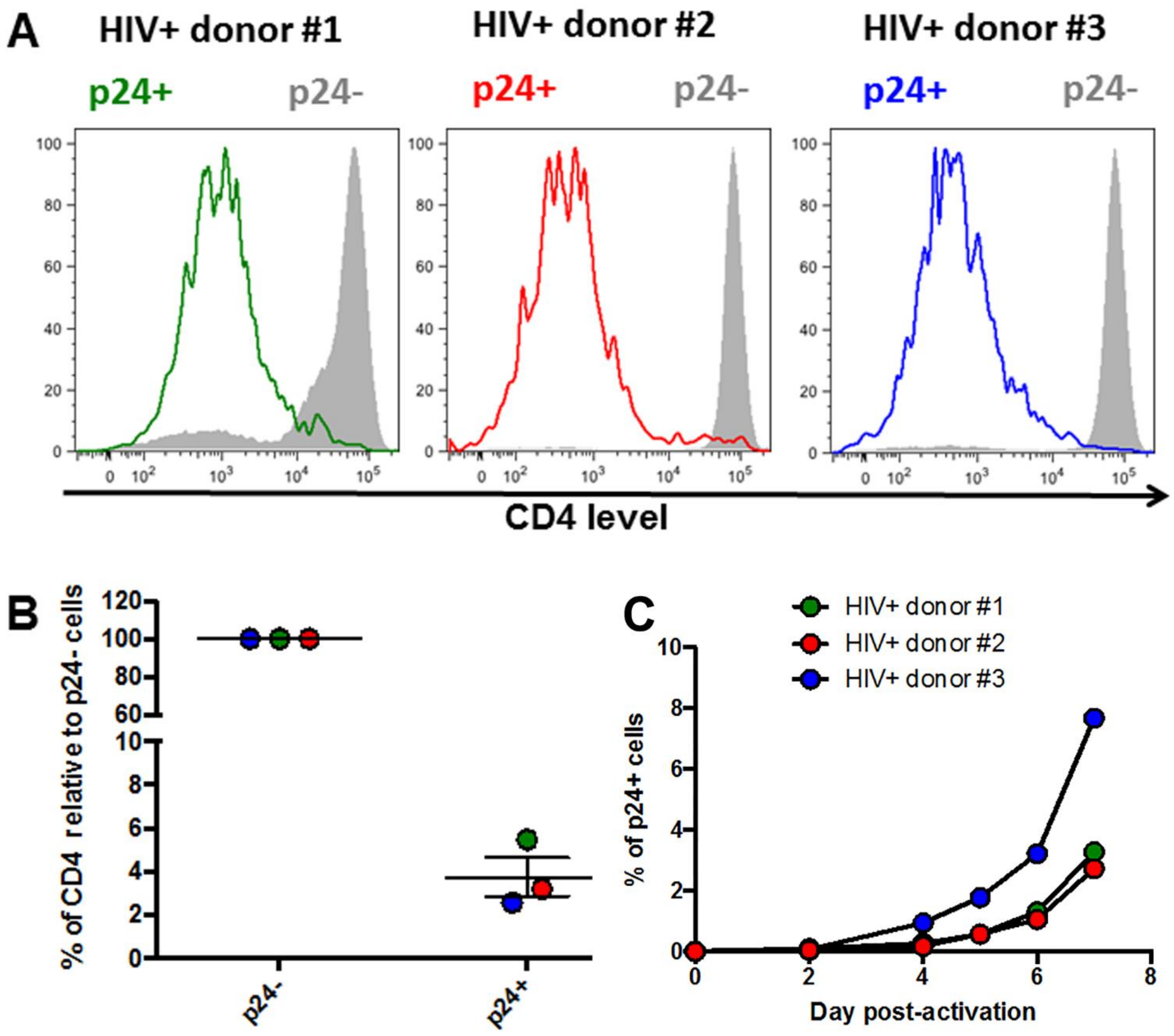

Figure S4: Level of cell-surface CD4 on endogenously-infected primary CD4+ T cells. Cell-surface staining of primary CD4+ T cells isolated from 3 HIV-1-infected individuals after activation, with an anti-CD4 mAbs. (**A**) Histograms depicting representative staining, (**B**) percentage of CD4 at the surface of HIV-1-infected (p24+) relative to uninfected (p24-) cells and (**C**) the kinetic of p24+ cells upon reactivation for the three HIV+ donors
